# Biotechnology and In Vitro Culture as an Alternative System for Secondary Metabolite Production

**DOI:** 10.3390/molecules27228093

**Published:** 2022-11-21

**Authors:** Marouane Mohaddab, Younes El Goumi, Monica Gallo, Domenico Montesano, Gokhan Zengin, Abdelhakim Bouyahya, Malika Fakiri

**Affiliations:** 1 Laboratory of Agrifood and Health, Faculty of Sciences and Techniques, Hassan First University of Settat, BP 577, Settat 26000, Morocco; 2 Polyyvalent Team in R&D, Higher School of Technology of Fkih Ben Salah, Sultan Moulay Slimane University, USMS, Beni Mellal 23000, Morocco; 3 Department of Molecular Medicine and Medical Biotechnology, University of Naples Federico II, via Pansini, 5, 80131 Naples, Italy; 4 Department of Pharmacy, University of Naples Federico II, via D. Montesano 49, 80131 Naples, Italy; 5 Department of Biology, Science Faculty, Selcuk University, Konya 42130, Turkey; 6 Laboratory of Human Pathologies Biology, Department of Biology, Faculty of Sciences, Mohammed V University in Rabat, Rabat 10106, Morocco

**Keywords:** secondary metabolites, cell culture, elicitor, biological effects

## Abstract

Medicinal plants are rich sources of bioactive compounds widely used as medicaments, food additives, perfumes, and agrochemicals. These secondary compounds are produced under stress conditions to carry out physiological tasks in plants. Secondary metabolites have a complex chemical structure with pharmacological properties. The widespread use of these metabolites in a lot of industrial sectors has raised the need to increase the production of secondary metabolites. Biotechnological methods of cell culture allow the conservation of plants, as well as the improvement of metabolite biosynthesis and the possibility to modify the synthesis pathways. The objective of this review is to outline the applications of different in vitro culture systems with previously reported relevant examples for the optimal production of plant-derived secondary metabolites.

## 1. Introduction

Plants can synthesize chemical compounds either as primary or secondary metabolites according to their biosynthetic pathways and their functions. The primary metabolites ensure the vital function of the plant. However, the process of secondary metabolites is not directly involved in plant growth and development. Even so, they have major roles in interactions with the environment as a means of defense and adaptation to environmental conditions [1].

The biosynthesis of secondary metabolites is based on geographical area, genetics, climate, and environmental conditions [2]. Under plant growth conditions, many secondary metabolites are amassed in distinct sites (vacuoles, specialized glands, trichomes, and sometimes only during certain developmental stages) to enable functional flexibility under the impact of environmental factors without influencing the cellular and physiological developmental pathways [3]. Indeed, these substances have high values for humans as pharmaceuticals, nutraceuticals, and cosmetics, making them targets for metabolic engineering [4]. Phytochemical investigations have identified an arsenal of secondary metabolites such as flavonoids, phenolic acids, nitrogen compounds, and terpenes [5,6].

The therapeutic effects of plants have been known since time immemorial [7]. These molecules, which are made by plants, are now utilized by the pharmaceutical industry from used vegetable raw materials [8,9]. While secondary metabolites exhibit various biological properties [10,11,12,13], their distribution is very limited compared to primary metabolites. Many of these compounds occur in very low quantities in nature [14,15], necessitating massive harvesting. This over-harvesting can threaten the biodiversity of the plants from which these secondary metabolites originate.

Biotechnological approaches can be considered a key and powerful substitute in the production of secondary metabolites coming from medicinal plants to support industrial production and reduce the overexploitation of natural resources [16]. However, cell, tissue, and plant organ culture techniques have been used for the production of these natural substances [17]. In this regard, effort has been made towards optimizing the culture conditions for the production of secondary metabolites, as well as manipulating the synthesis of these phytoconstituents through the application of different technological approaches including cell line selection, elicitation, and precursor feeding [18]. These efforts have been carried out to increase secondary metabolite production to meet the demand of the pharmaceutical industry and to conserve natural sources [18,19,20,21,22].

Several extraction methods can be applied, depending on the physicochemical nature of these compounds of interest [23]. These methods can be conventional or modern. Conventional methods are generally based on the extraction potential of the different solvents used before applying heat to them and/or mixing the solvents to obtain bioactive compounds, such as Soxhlet extraction, maceration, and hydrodistillation [24,25,26], while modern extraction techniques allow for shorter extraction time and reduced solvent consumption [27]. New extraction methods, including ultrasonic-assisted extraction [28,29,30], supercritical fluid extraction [29,30,31], and accelerated solvent extraction [32], are fast and efficient for extracting chemicals from plant matrices. In addition, in situ extraction is considered an efficient method to recover secondary metabolites; moreover, it allows both to improve the yield of the product and to orient the secondary metabolite pathway’s in vitro culture system [33,34,35]. As the results revealed, the use of perfluorodecalin in the in situ extraction system improved the performance of the cells’ culture as well as increased the production of targeted molecules [36,37]. The choice of an appropriate extraction method should be an essential consideration depending on the study objective, as the process of the extraction may fully influence the chemical composition and therefore the biological activity of the extract [38].

Plant extracts constitute a mixture of bioactive or phytochemical compounds of several polarities, and their separation is an important challenge that leads to identification and characterization processes [39]. In general, high-performance liquid chromatography (HPLC) and gas chromatography (GC) coupled with mass spectrometry (MS) or nuclear magnetic resonance spectroscopy (NMR) are widely used to characterize and quantify secondary metabolites in plant extracts.

For a long time, herbal treatments have been widely used for primary healthcare needs. Through time, and with progress in the field of pharmacopy, synthetic drugs have gradually started to be used instead of natural drugs, regardless of the side effects of the synthetic components [40]. Moreover, these natural products have lower hydrophobicity and higher stereochemical content than synthetic products [41]. Structural features of natural compounds can be effectively incorporated into synthetic drugs to increase chemical diversity, and molecular complicity is an important feature for drugs [42], as molecular complexity has been correlated with biological activity [43]. Indeed, in recent years, approval of synthetic drugs has declined substantially [40,43]. So far, many successes have been registered in the discovery of new active molecules in natural compounds. Some of these molecules have become medicines or new paths of inspiration in finding new ones [44]. On the other hand, medicinal plants and their natural products are still the best pharmaceutical lead and offer an opportunity to discover new structures effective in a variety of human diseases [38,44]. However, such property may threaten the biodiversity of these medicinal plants due to overexploitation and unsustainable harvesting techniques [45].

In addition, plant biotechnology has offered alternative ways to access and explore this chemical diversity through different in vitro culture techniques to produce natural products for the pharmaceutical industries [46,47,48]. The cell culture technique can be used as a platform for the production of high-value secondary compounds [46,48,49,50]. Different biotechnology approaches represent a beneficial alternative for the production of secondary metabolites under highly controlled conditions [51,52]. Therefore, in vitro culture techniques such as plant organ culture provide plant material as a source of natural products [38]. Multiple strategies using cell culture systems have been widely studied in the context of improving the production and manipulating the flow of the biosynthesis of desired secondary metabolites [46,53].

Plant cell and tissue culture offer an opportunity for the propagation of plants as well as the production of phytochemicals [54]. Many plant species can be regenerated in vitro through several approaches started by explants. Any part of the plant, such as meristems, nodes, leaves, stems, roots, buds, embryos, etc., can be used for a limitless multiplication of a plant and the production of bioactive compounds under sterile conditions [48,55,56,57]. Due to its various advantages, in vitro culture has been used as a powerful strategy for the production of secondary metabolites [22,58]. In this review, we highlight biotechnological approaches as promising strategies for the synthesis and improve secondary metabolites in medicinal plants.

## 2. Plant Secondary Metabolites

Plant Secondary metabolites (PSMs) are low-weight molecules synthesized by the plant to protect itself against potential enemies, including pathogens and herbivore attacks. Even abiotic factors can affect the biosynthesis of secondary metabolites [59,60].

Due to their excellent biological activity, PSMs have been broadly used for centuries as an important resource for traditional medicine, perfumes, and industrial raw materials [61]. Subsequently, they have been widely applied as valuable compounds such as pharmaceuticals, cosmetics, and bio-pesticides [4,51,61,62]. PSMs have contributed greatly to the importance and commercial values of plants [63].

**Figure 1:**
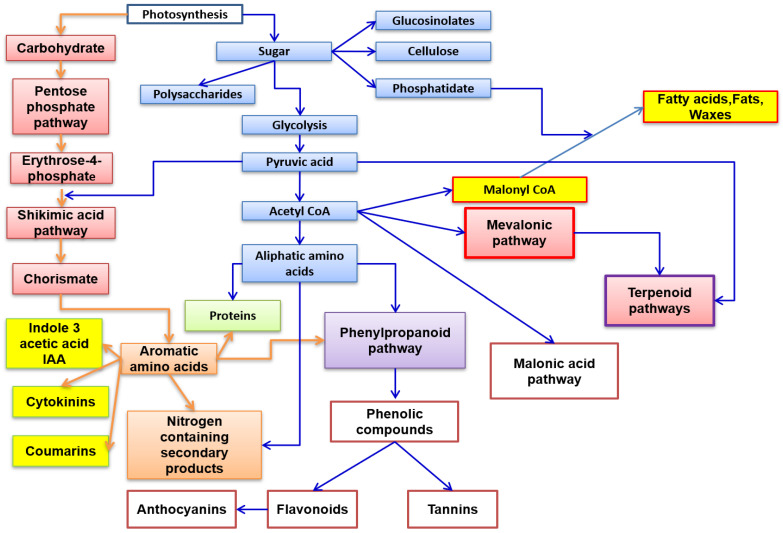
Principal biosynthetic pathways of major secondary metabolite plants’ classes.

Phytochemical studies have identified an arsenal of secondary compounds such as flavonoids, phenols, nitrogen compounds, and terpenes [5,6]. The more detailed biosynthetic pathways of these metabolites are beyond the scope of this review. Thus, a preview of the various biosynthetic pathways is represented in Figure 1.

## 3. Micropropagation as a Tool for the Production of Secondary Metabolites

Micropropagation is the reproduction of plants in vitro which leads to the multiplication of genetically identical copies of the parent plant asexually. Micropropagation offers the possibility of producing a limitless number of plants. Currently, this technique is applied for clone selection and rapid biomass production in several organizations or establishments for the large-scale production of higher plants.

In vitro propagation has become a crucial method for the mass production of medicinal plants and various protocols of the micropropagation of numerous medicinal species that have been successfully achieved either by organogenesis [64,65,66,67,68] or by somatic embryogenesis [69,70,71]. The micropropagation of many medicinal species has been revealed to be similar and with a little variation in their phytochemical content [72].

Organogenesis is a micropropagation way that consists in the development of organs derived from cells or tissues. Plant regeneration through organogenesis involves specifically the induction and development of a shoot from an explant which is then transferred to a different medium for root induction [73]. Several studies have demonstrated that a successful application of organogenesis on medicinal plants can be achieved by the correct establishment of the medium components and the selection of an adequate explant under highly controlled conditions (Table 1).

Somatic cells can produce somatic embryos, which are similar to zygotic embryos, through a process called somatic embryogenesis. These somatic embryos can be developed into seedlings in an appropriate medium [81]. Plant regeneration via embryogenesis occurs in two steps: the callus is grown on an auxin-rich embryogenic induction medium, sometimes combined with cytokinins, and is then transferred to an auxin-free medium, which results in the formation of mature embryos [82]. The embryonic-like structure can be produced either directly on the explant or indirectly from the callus or cell suspension culture (Table 2). This technique has also allowed genetic, morphological, and physiological manipulations to be performed [83].

Micropropagation could be an attractive commercial activity for the production of high-quality plants and offers advantages over conventional propagation practices [88]. Thus, in vitro propagation is a sustainable alternative to the large-scale production of medicinal species with economic value. Castilho et al. [80] allowed the use of an automated micropropagation system using bioreactors for industrial plant propagation as a possible way to reduce micropropagation costs [89]. This can provide a means of supplying plant material capable of providing plant material that is able to produce phytocompounds [19,38,48,90] throughout the year without seasonal constraints [16].

## 4. The Importance of Cell and Suspension Culture in the Production of Plant Secondary Metabolites

The evolution of biotechnology, in particular plant cell culture methods, should provide new means for the commercialization of plants and their chemical compounds. These new technologies will expand and enhance the use of plants as valuable resources of pharmaceutical compounds. Plant cell cultures have attracted considerable interest in the industrialization of secondary metabolite production [91,92].

In vitro production of secondary metabolites requires the aggregation of cell biomass for the synthesis of secondary metabolites [93]. Under in vitro conditions, plant cells that induce callus formation through a high concentration of auxins or with the coordination of auxin and cytokinin are frequently used [46]. Subsequently, callus can be used to develop a suspension culture for the production of secondary metabolites [20,22,94]. In addition, the immobilization of the cell system of hairy root plants is an efficient technique to produce relevant bioactive compounds [34,35].

Plant cells, as defense mechanisms, produce secondary metabolites [16]. In this light, the strategy to improve the synthesis of secondary metabolites, elicitation, is through the application of agents that trigger the defense response. Hence, there have been several authors who have illustrated the application of elicitors to enhance the production of secondary metabolites [95,96,97,98,99]. Similarly, plant growth regulators are known for their ability to regulate the production of secondary metabolites [100,101,102]. Several studies confirmed that phytohormones increase the production of secondary metabolites [101,103,104] (Table 3).

The in vitro culture of a *Fritillaria unibracteata* bulb by [120] confirmed that the growth rate of the in vitro culture was faster than under natural conditions. The alkaloid and microelement content of the in vitro cultured bulbs were higher compared to the wild bulbs. Moreover, for the in vitro culture of *Clinacanthus nutans* leaves, [121] remarked that the phenolic content and antioxidant activities were improved. Moreover, fungal elicitors have been used to improve the production of secondary metabolites in *Hybanthusenneaspermus* [122]. Furthermore, a cell suspension culture inclusion of α-Naphthalene acetic acid (NAA) and Kinetin (KIN) from *Eysenhardtiaplatycarpa* showed a significant biomass accumulation, as well as the dichloromethane extracts of the suspension which contains phenolic components and flavonoids with remarkable antifungal activity [112]. Phyllanthusol A was produced by callus culture in MS medium with NAA and BA [115]. Indeed, [117] remarked that callus can accumulate the same secondary metabolites (53 metabolites were identified) produced in the leaves (47 compounds in leaf extracts) of *Rosmarinus officinalis*.

## 5. Bioreactors: System for Large-Scale Production

The synthesis of secondary metabolites through in vitro culture has led to the concept of bioreactors for the large-scale production of natural compounds in recent years [50,123,124]. Moreover, bioreactors are autonomous systems that have a sterile environment, control, and which provide homogeneous culture conditions in terms of pH, aeration and temperature, and agitation, as well as liquid and air inlet and outlet channels for the massive multiplication of cells, tissues, or somatic embryos [125,126]. Reviews published [127,128,129] contain schematics of different types of bioreactors. Therefore, Bioreactors are engineered systems that can support the biological condition and aim of the realization of aerobic or anaerobic biochemical processes. This means that bioreactors can replace the conventional methods of in vitro culture [130,131].

Bioreactor culture has led to the production of many products such as shikonin, a rich reddish-purple pigment used in lipsticks [132], ginsenosides used as additives and bleaching substances [53,133], paclitaxel (as well as the anti-cancer drug) [134], and in food applications [135]. In addition, bioreactor production has been reported by several authors [136,137,138,139,140,141,142].

*Panax Ginseng* suspension culture in the bioreactor enhanced biomass accumulation as well as ginsenosides (5.4 mg/g) [136]. Similarly, ginseng culture treated with salicylic acid led to an accumulation in total phenolic (62%), flavonoids (88%), and ascorbic acid (55%) [142]. Somatic embryos can be grown in bioreactors as a source of raw materials since they can accumulate secondary metabolites [141]. The cultivation of adventitious roots of *Hypericum perforatum* in a bubble bioreactor containing MS half-strength medium with 0.1 mg/L Kn and 1 mg/L IBA accumulated total phenolics (35.01 mg/g DW), flavonoids (0.97 mg/g DW), and hypericin (1.389 mg/g DW) [143]. The highest production of flavonoids from *Gynuraprocumbens* was obtained in the temporary immersion bioreactors under the combined treatment of 15 min immersion frequency every 12 h in MS medium with IAA and BA [144]. In vitro shoot culture of *Verbena officinalis* was grown in temporary immersion bioreactors complemented with 4.92 µM IBA and produced large amounts of biomass with increased levels of essential oils [128,137]. Single-use bioreactors are suitable systems to increase and control the microenvironment culture. In this approach, the hairy root culture of *Ringeragraeca*, supported by the WAVE 25 bioreactor system, exhibited a strong increase in fresh biomass (more than 800%) and a very high yield of naphthoquinone (Wierzchowski). Moreover, the culture of the cambial meristematic cells of *O. basilicum* in wave-mixed disposable bioreactors was shown to produce the highest yield of triterpenoids (oleanolic acid = 3.02 ± 0.76 mg/(l × d) and ursolic acid = 4.79 ± 0.48 mg/(l × d)), 1.75-times higher than the shake [130].

Thus, bioreactors could improve the efficiency of the process for more valuable plant-derived products and lead to a new wave of industrial production.

## 6. Elicitation of In Vitro Products

The use of substances that trigger the defense response of plants and cells in vitro culture is considered an excellent biotechnological method for the production of secondary compounds [16,145]. An elicitor is defined as a factor or element that, once introduced or modified in an in vitro culture, increases the biosynthetic capacity of secondary metabolites [98]. Generally, there are two types of elicitors: biotic and abiotic. Both of them have been well detailed in several reviews [56,95,98,146,147,148,149,150].

Adding an eliciting agent can improve the production of the secondary metabolites of medicinal plants by in vitro culture. Many fields can use this approach, which allows the production of high-value bioactive compounds such as pharmaceuticals, food, and cosmetics [151]. The quantity and quality of the obtained metabolites can be greatly influenced by various parameters such as the nature of the elicitor, its concentration, and the exposure time, Table 4 [152,153,154,155,156,157,158].

Abiotic elicitors have wide effects on the production of secondary metabolites [159]. For example, Chavan et al. [160] reported that the application of jasmonic acid (75 µM) in callus cultures in *Salacia chinensis* improved the total phenolic, flavonoid, and mangiferin contents for the same application, which revealed the highest antioxidant potential. Moreover, Mahendran et al. [161] documented that Gymnemasylvestre cell suspension culture with 20 μM sodium nitroprusside treatments revealed the highest accumulation of deacylgymnic acid and XVII gymnemic acid. Furthermore, the cultivation of *Carum copticum* under salt stress enhanced the phenolic content accumulation and antioxidant activity [162]. Similarly, elicitation with nanoparticles could enhance the production of the secondary metabolites of *Fagonia indica* in callus cultures [163]. In the suspension culture of *Lonicera japonica* Thun, a combination of 200 μM methyl jasmonate, 50 μM salicylic acid, and 2 h d-1 Ultraviolet B radiation, improved the synthesis of the chlorogenic acids and showed a high antioxidant capacity compared to untreated control and field-grown buds [164]. Açıkgöz, [165] demonstrated the stimulatory effects of CdCl_2_ and AgNO_3_ on the accumulation of bioactive components in *Ocimum basilicum* cell suspension cultures.

Biological substances such as polysaccharides and microbial compounds can be used as biotic elicitors [159]. In the callus cultures of *Lepidium sativum*, the application of chitosan (250 mg/L) increased the concentration of lepidin and total phenolic compounds by 19.87 times compared to the control value [166]. Elicitation by chitosan in *Silybum marianum* cell suspension increased the production of silymarin and revealed high antioxidant and anti-inflammatory activities [167]. Furthermore, Farhadi et al.’s [168] cell suspension culture of *Corylus avellana* with a fungal elicitor application enhanced the biosynthesis of paclitaxel. Treatment with an aqueous extract of *Spirulina platensis* increased the production of linalool in *Lavandula officinalis* [169]. Yeast extract increased chicoric and rosmarinic acid content in suspension cultures of *Ocimum basilicum* [165]. Salehi et al. [170] reported the positive effects of fungal elicitors on paclitaxel production in the cell suspension culture of *Corylus avellana*. Moreover, [171] reported that introducing elicitors from endophytic fungi (*Chaetomium sp*.) into a culture of adventitious roots of *Panax ginseng* had a significant increase in ginsenosides (56.29 mg/g) relative to the controls (17.56 mg/g).

Further studies on the elicitation of hairy root cultures [172,173,174,175,176] highlighted the potential to produce higher amounts of secondary metabolites. Hashemi and Naghavi [172] demonstrated elicitation in the hairy root culture of *Papverorientale* with methyl jasmonate and salicylic acid, which resulted in the regulation of the expression of genes in the morphine pathway; moreover, the elicitation of methyl jasmonate (MJ) improved the synthesis of thebaine (3.08 mg/g), morphine (5.38 mg/g) and codeine (2.57 mg/g). Moreover, the results demonstrated that the elicitation by chitosan (200 mg/L) in the hairy culture of *Psammosilenetunicoides* a produced a 4.55-fold increase in total saponin accumulation for nine days, and that the yields of quillaic acid, gypsogenin, and gypsogenin-3-*O*-*β*-D-glucuronopyranoside were significantly increased after the chitosan treatments.

**Table 4:** Some application of abiotic and biotic elicitors in the production of plant secondary metabolites.

Plant Species	Elicitor Factor	Culture System	Product	Key Findings	References
**Abiotic elicitors**					
***Chelidonium majus***	Methyl jasmonate (MJ) Salicylic acid (SA)	Cell suspension culture	Chelidonine, sanguinarine	Elicitation stimulated the expression of genes in the benzophenanthridine alkaloid biosynthetic pathway.	[177]
***Ocimumbasilicum***	Copper oxide (CuO)	Callus culture	Rosmarinic acid, chicoric acid, eugenol	Elicitation by nanoparticles stimulated the biosynthesis of the secondary metabolite.	[178]
***Ocimumbasilicum***	Salicylic acid (SA) + light regimes	Callus culture	Rosmarinic acid, chicoricacid, cyanidin, peonidin	Continuous light with SA increased the content of phenolic compounds and flavonoids, also antioxidant activity.	[178]
***Coelogyne ovalis***	Salicylic acid (SA)	Tissue culture	Flavonoids, anthocyanins, phenolic compounds	Elicitation stimulates chalcone synthase expression and secondary metabolites production.	[179]
***Papaver orientale***	Methyl jasmonate (MJ), salicylic acid (SA)	Hairy root culture	Thebaine, morphine, codeine	Expression of morphinan biosynthetic genes was significantly upregulated with MJ and SA. MJ and SA elicitation enhanced thebaine, morphine, and codeine biosynthesis.	[172]
***Crocus sativus***	Ultrasonic waves	Cell suspension culture	Safranal, crocin	Ultrasonic treatment acted as an effective mechanical stimulus on the production of secondary metabolites in suspension cultures.	[180]
***Gymnemasylvestre***	Sodium nitroprusside (SNP)	Cell suspension culture	Deacylgymnemic acid, gymnemagenin, gymnemic acid XVII	Significant improvement in the content of gymnemic acids in cell suspension cultures of *G. sylvestre.*	[161]
***Momordica charantia***	Silver nanoparticles (AgNPs)	Cell suspension culture	Hydroxybenzoic, hydroxycinnamic	The significant increase in bioactive compounds as well as pharmacological activities was enhanced by the application of elicitation.	[181]
**Biotic elicitors**					
***Corylus avellana***	Chaetommiuglobosum	Cell suspension cultures	Paclitaxel	Increased extracellular portion of paclitaxel (44.0%).	[170]
***Bletilla striata***	Byssochlamys spectabilis	Tissue culture	Total phenolic content	Increased total phenolic compounds.	[182]
***Panax ginseng***	Aspergillus niger	Adventitious root culture	Ginsenosides	*A. Niger* triggered the defense response of plants and enhanced the accumulation of nitric oxide (NO), SA, and JA. Significantly upregulated the gene expression of terpenoid biosynthesis.	[183]
***Panax ginseng***	Alternaria panax	Adventitious root culture	Ginsenosides	Nitric oxide (NO), putrescine (Put), and hydrogen peroxide (H_2_O_2_) are involved in regulating ginsenoside synthesis in fungal elicitor-treated Adventitious root of *P. ginseng*.	[184]
***Trichosanthescucumerina***	Chitosan	Callus and suspension culture	Bryonolic acid	Callus and suspension cultures presented higher levels of Bryonolic acid than the natural roots ones.	[185]
***Psammosilenetunicoides***	Chitosan	Hairy root culture	Quillaic acid, gypsogenin, gypsogenin 3-*O*-β-D-glucuronopyranoside	Chitosan elicitor promotes triterpenoid saponin biosynthesis by enhancing antioxidant activities and differential gene expression.	[175]
***Iberis amara***	Chitosan	Cell suspension culture	Total phenol, flavonoid, flavonol, anthocyanin	Chitosan elicitor promotes phenolic compounds’ biosynthesis without genetic modifications in medicinal herbs.	[186]
***Plumbago zeylanica***	Chitosan and yeast extract	Root callus	Plumbagin	Increase of 12.08-fold plumbagin content compared to control.	[187]

## 7. Conclusions and Perspectives

Medicinal plants represent an impressive reservoir of bioactive compounds with several pharmacological properties. Biotechnological approaches and in vitro culture constitute a precious, sustainable, and ecological alternative for the production of these bioactive compounds to reduce the use of chemically synthetic compounds while decreasing the overexploitation of natural resources. In this respect, the synthesis of secondary metabolites by in vitro culture has experienced several successes in a variety of culture systems. The industrial production of secondary metabolites is not totally developed because of the low yields of the compounds targeted. Furthermore, the biosynthetic pathways of secondary metabolites are not fully characterized, nor is the epigenetic control of the biosynthesis of these compounds in long-term culture [188]. However, further studies are required to comprehend the biosynthetic pathways and the epigenetic mechanisms that regulate the biosynthesis of secondary metabolites to guarantee targeted production with a high and stable yield of the secondary compounds wanted.

## Figures and Tables

**Table 1:** Micropropagation of medicinal plants by organogenesis methods.

Plant	Explant Source	Shoot Multiplication		Rooting		Phytochemical Analysis	Key Findings	References
		MS Medium	Phytohormone	MS Medium	Phytohormone			
***Zingiber officinale Roscoe***	Rhizome sproutedbud	solid	Zeatin (10 µM)	solid	NAA (7.5 µM)	Flavonoids and phenolic acids	The total content of phytochemical components is not very different from those of conventionally propagated plants.	[74]
***Plectranthusamboinicus***	Axillarybuds	semi-solid	BAP (0.4 mg/L)	semi-solid	Without PGR	Carvacrol γ-Terpinene	Essential oil yield was improved with a higher quantity of chemical compounds in vitro cultures. The in vitro regeneration was chemically true to the parent plant type.	[64]
***Lavandula coronopifolia***	Shoot tips	solid	BA (0.5 mg/L)	solid	IBA (10 mg/L)	Caffeic acid androsmarinic acid	Micropropagation was regenerated from plants with genetic fidelity to the parent plant. A remarkable difference in the chemical profiles of the in vitro culture and the wild-type plants.	[75]
***Tanacetum vulgare***	Shoot tips	solid	without PGR	liquid half-strength	Without PGR	Monoterpenes Sesquiterpene Chlorogenic acid 3,5-*O*-Dicaffeoylquinic acid	Spontaneously rooted seedlings at the time of propagation. Terpenes are the most abundant in essential oils. In vitro grown roots are richest in 3,5-O-dicaffeoylquinic acid.	[76]
***Cannabis sativa***	Nodal segments	solid	mT (2 µM)	solid	mT (2 µM)	Cannabinoids	Rooting was performed on the same propagation medium. Auxin was not necessary for root induction. cannabinoid level in the micropropagated plants is comparable to the mother plant. In vitro propagated plants are identical to the mother plant.	[77]
***Eryngiumalpinum***	Shoots	solid	BAP, IAA, and GA3 (each 1.0 mg/L)	__	__	Phenolic acids and flavonoids	The solid MS medium with BAP, IAA, and GA3 (each 1.0 mg/L) is the optimal system for micropropagation and accumulation of phenolic acids and flavonoids. An important variability in phytochemicals between the intact plant and different in vitro culture.	[6]
***Spiraeabetulifoliasubsp. aemiliana***	Axillarybuds	solid	S1 = BAP (1.0 μM) S2 = (BAP 5.0 μM) + (NAA 1.0 μM)	half-strength	S1 = S2= IBA (0.1 µM)	Phenolic acids and flavonoids	Many differences in chemical profile between in vitro culture and intact plants. Interpopulation genotypic differences in the activity of morphogenic processes have been identified in *S. betulifolia* in vitro culture.	[78]
***Salvia sclarea***	Nodal segments	solid	mT (2.0 mg/L) + IAA (0.2 mg/L)	solid	NAA (1.0 mg/L)	A multitude of secondary metabolites	High genetic stability of micropropagated plants. N-alkanes, tetradecanal, octadecanal, and hentriacontane are the major components from micropropagated plants. PGRs have caused variability in the content of secondary metabolite.	[79]
***Lippiaoriganoides***	Nodal segments	solid	KIN (4.6 μM)	solid	KIN (2.3 μM)	Myrcene, p-cymene, γ-terpinene, linalool, thymol, carvacrol and (E)-caryophyllene.	The presence of PGR changed the chemical profile of the volatile organic compound.	[80]

Murashige and Skoog (MS), 6-benzylaminopurine (BAP), α-Naphthalene acetic acid (NAA), Benzyl adenine (BA), indole-3-acetic acid (IAA), Indol-3-butytic acid (IBA), Gibberellic acid (GA3), Kinetin (Kin), meta-Topolin (mT), plant growth regulator (PGR).

**Table 2:** Micropropagation of medicinal plants by somatic embryogenesis (SE).

Family	Plant	Explant Source	Phytohormone (mg/L) for Induction SE	Basal Medium	Somatic Embryogenesis		References
					Direct	Indirect	
**Apiaceae**	*Ferulajaeschkeana*	Petiole	2,4-D (4.0)	MS	-	X	[84]
**Asteraceae**	*Seriphidiumherba-album*	Leaves	2,4-D (1.5) + BA (0.5)	MS	-	X	[85]
**Fumariaceae**	*Lamprocapnosspectabilis*	Leaves	2,4-D (0.5) + BA (0.5)	½ MS	-	X	[86]
		Petioles	PIC (1.0) + BA (0.5)				
**Plantaginaceae**	*Digitalislanata*	Leaves	2,4-D (1.0) + Kin (1.0)	MS	-	X	[87]
			IBA (2.0) + Kin (2.0)		X	-	
		Root	IBA (2.0) + Kin (2.0)		X	-	

Murashige and Skoog (MS), 2,4-dichlorophenoxyacetic acid (2,4-D), Benzyl adenine (BA), Indol-3-butytic acid (IBA), Kinetin (Kin), Picloram (PIC).

**Table 3:** List of some applications of cell and suspension culture in the production of secondary metabolites.

Plant Species	Active Ingredient	Culture Condition (MS Medium)	Culture Type	References
***Ageratinapichinchensis***	Artemesinol	NAA + KIN	Suspension	[105]
***Anethum graveolens***	Carvone	BA + NAA + SA	Suspension	[106]
***Camellia sinensis***	Catechin	BAP + 2,4-D + Ph (phenylalanine)	Callus	[107]
***Capparis spinosa***	Rutin	B5 medium + 2,4-D + BAP + MeJA + SA	Callus	[108]
***Carallumatuberculata***	Total phenolics	MS + 2,4-D + BAP + AgNPs(silver nanoparticles)	Callus	[109]
	Total flavonoid			
***Cayratia trifoliata***	Stilbenes	NAA + KN + MeJA	Suspension	[110]
***Cupressus sempervirens***	RutinQuercitrin	BA + NAA + GA3	Callus	[111]
***Eysenhardtiaplatycarpa***	Total phenolics	NAA + KIN	Suspension	[112]
***Gardeniajasminoides***	Rutin	TDZ	Callus	[113]
***Gymnemasylvestre***	Gymnemic acid	2,4-D + BA + MeJA	Suspension	[114]
***Phyllanthus acidus***	Phyllanthusol	NAA + BA	Callus	[115]
***Pluchealanceolata***	Quercetin	NAA + BAP	Callus	[116]
***Rosmarinus officinalis***	Flavonoid	2,4-D + BAP	Callus	[117]
	Terpenoids			
***Ocimumbasilicum***	Rosmarinic acid	KIN + NAA + Sorbitol	Suspension	[118]
	Chicoric acid			
	Rutin			
	Linalool			
	Methyl chavicol			
***Labisia pumila***	Total phenolics	2,4-D + Zea	Callus	[119]
	Total flavonoid			

Murashige and Skoog (MS), Gamborg’s (B5), 2,4-dichlorophenoxyacetic acid (2,4-D), 6-benzylaminopurine (BAP), α-Naphthalene acetic acid (NAA), Benzyl adenine (BA), Kinetin (Kin), Gibberellic acid (GA3), Thidiazuron (TDZ), Zeatin (Zea), MeJA (methyl jasmonate), SA (Salicylic acid).

## Data Availability

Not applicable.
